# Research gaps in sentinel lymph node biopsy in breast cancer

**DOI:** 10.1093/oncolo/oyae133

**Published:** 2024-06-05

**Authors:** Omar Hamdy

**Affiliations:** Surgical Oncology Department, Mansoura University Oncology Centre, Mansoura, Egypt

## Abstract

This commentary focuses on 5 questions that need to be answered when considering sentinel lymph node biopsy in breast cancer, aiming to highlight these issues and provide researchers with ideas to resolve.

Sentinel lymph node biopsy (SLNB) in breast cancer is now exceeding its known role as the standard of care in axillary lymph node (LN) staging to replace standard axillary lymph node dissection (ALND) in selected cases of node-positive breast cancer. This role is expanding year by year and the threshold to perform SLNB has become steadily lower, and the restrictions looser. It is now clear that the goal of axillary surgery is mainly diagnostic rather than therapeutic. It helps properly stage the disease and outline the treatment plan, and on the other hand, axillary evacuation has proved not to be of the previously thought therapeutic importance.^1-4^

Despite answering many questions regarding its efficacy and safety, there are still gray zones and points that need further research to be illuminated.

In this commentary, I focus on 5 questions that still need to be answered, aiming to highlight these issues and provide researchers with ideas to help fill the knowledge gaps.

## What should the breast surgeon do if he has positive one out of one or 2 out of 2 sentinel nodes?

The ACOSOG Z0011 trial was a practice-changing study that paved the way to limiting the role of ALND in patients who met all of the following criteria: only one or 2 positive sentinel nodes, small tumors, did not receive neoadjuvant therapy, and underwent breast-conserving therapy with planned postoperative axillary irradiation.^5,6^ Recently, the guidelines were expanded to include patients who underwent mastectomy as well.^7,8^ Many surgeons worldwide have adopted the Z0011 policy and omit ALND when there are one or 2 positive LNs out of 2 or more total nodes excised.^9-11^

However, breast surgeons may face ambiguous situations with no clear answers based on existing evidence, eg, what if a patient has positive one out of one or 2 out of 2 dissected sentinel nodes? Should the surgeon stick literally to the Z0011 criteria and omit ALND accepting the risk that there could be additional positive axillary nodes, or should he pursue ALND carrying the risk that all the excised LNs may be negative for malignancy and exposing the patient to unnecessary complications of ALND?

Further research should be conducted to include this category of patients and compare the surgical and oncological outcomes in patients with 1/1 or 2/2 positive LNs on SLNB when they undergo completion versus omission of ALND.

## What should the decision regarding ALND be in patients who received neoadjuvant therapy with nodal conversion in institutions lacking radioactive materials or in patients in whom pretreatment lymph node clipping was not performed and the retrieved sentinel nodes are less than 3?

The management of the axilla after neoadjuvant therapy has witnessed many paradigm shifts, especially in those who converted from node-positive to node-negative disease and especially in the last decade. Because the main concern is the relatively high false negative rate in these patients, some prerequisites are usually advised to ensure the oncological safety of performing sentinel node biopsy rather than ALND. These prerequisites include the use of a dual mapping technique (radioactive isotope + blue dye), retrieval of 3 or more lymph nodes, and targeted axillary dissection, which entails clipping of the positive nodes before neoadjuvant therapy then removal of the sentinel nodes in addition to the clipped nodes at the time of surgery.^7,12^

In practice, nuclear medicine facilities are not available in all institutions worldwide, especially in the Eastern world.^13,14^ Also, the surgeon may meet a patient with positive nodes before treatment who did not undergo clipping. Last, retrieval of 3 sentinel nodes at the time of surgery does not usually occur. The options here are either SLNB, which carries the risk of a relatively high false-negative rate or ALND, depriving these patients of the benefit of nodal downstaging achieved by neoadjuvant therapy.

In the last 2 years, this issue has received more attention, with studies comparing outcomes for patients who underwent sentinel node biopsy alone and confirming the non-inferiority of SLNB alone to ALND, despite the former’s relatively high false-negative rate.^15-17^ Further supporting studies are needed to allow updating international guidelines.

## Is it always practical to clip all positive lymph nodes before neoadjuvant therapy?

Another issue related to targeted axillary dissection is lymph node clipping before neoadjuvant therapy.^18^ If the initial radiologic evaluation reveals 3 or more malignant-looking lymph nodes, is it practical or even possible to put a clip in all the malignant-looking lymph nodes? If a patient has 5 malignant-looking axillary lymph nodes, should we clip them all? Will the cost of the titanium clips and the radiologists’ capabilities all over the world support such a strategy? And even if it were possible, should we remove all these lymph nodes in addition to the sentinel nodes after neoadjuvant therapy? This may cause lymphatic damage equal to that caused by ALND.

## Should we proceed to ALND in N2-3 patients even after an excellent clinical response?

It is accepted as of this writing that patients with heavy nodal infiltration before neoadjuvant therapy (N2-3) should undergo ALND regardless of the response to treatment.^19,20^ However, this may be an unfair practice, especially for patients with triple negative disease or HER2-positive disease receiving neoadjuvant chemotherapy and dual anti-HER2 therapy, respectively, in which a nodal complete pathological response is more likely to be achieved.^21,22^

Further research should be conducted with a special focus on this category of patients who are usually considered a high-risk group for regional recurrence and distant metastasis.

## How should the breast surgeon proceed after neoadjuvant therapy in the initially node-positive axilla with post-treatment axillary nodes having indeterminate sonographic criteria?

Regardless of the axillary staging procedure used after neoadjuvant therapy in those with initially node-positive disease, breast surgeons repeatedly face a confusing situation when the axillary lymph nodes show indeterminate ultrasonographic criteria that cannot lead to a certain decision whether the lymph nodes are still malignant or not.^23^

Until now, the standard of care for node-positive disease that remains positive after neoadjuvant therapy is ALND. For those who converted to negative nodes,^15,18^ the standard is targeted axillary dissection (or SLNB). But what about those who have an uncertain nodal status after neoadjuvant therapy? Should the surgeon proceed with ALND, or should he consider the nodes to be negative? Or should surgery be postponed for an ultrasound-guided biopsy from the most significant lymph node?

These 5 questions represent gaps that lack sharp answers to frequently faced situations in surgical oncology practice. The optimal oncological philosophy is that which aims to achieve the best survival outcomes and improve quality of life without affecting oncological safety or putting patients at risk.

## References

[1] Hamdy O , Farouk O, El-Badrawy A, Denewer A, Setit A. Sentinel lymph node biopsy in breast cancer—an updated overview. Eur Surg. 2020;52(6):268–276. 10.1007/s10353-020-00665-w

[2] Kaushik M. Evolution of sentinel lymph node biopsy. In: Chintamani, editor. Sentinel node biopsy in breast cancer. New Delhi: Springer India; 2023:1–7. 10.1007/978-81-322-3994-9_1

[3] Peristeri DV , Harissis HV. Axillary lymph node dissection vs sentinel biopsy only among women with early-stage breast cancer and sentinel node metastasis: a systematic review and meta-analysis. Breast J. 2021;27(2):158–164. 10.1111/tbj.14140 33368762

[4] Giannakou A , Gooch J, Gergelis KR, Weiss A. De-escalation of axillary surgery in patients outside Z0011 criteria. Surgery. 2023;174(2):416–418. 10.1016/j.surg.2023.03.024 https://www.sciencedirect.com/science/article/pii/S0039606023001708 37156648

[5] Giuliano AE , McCall L, Beitsch P, et al. Locoregional recurrence after sentinel lymph node dissection with or without axillary dissection in patients with sentinel lymph node metastases: the American College of Surgeons Oncology Group Z0011 randomized trial. Ann Surg. 2010;252(3):426. 10.1097/SLA.0b013e3181f08f32 20739842 PMC5593421

[6] Giuliano AE , Ballman KV, McCall L, et al. Effect of axillary dissection vs no axillary dissection on 10-year overall survival among women with invasive breast cancer and sentinel node metastasis: the ACOSOG Z0011 (Alliance) randomized clinical trial. JAMA. 2017;318(10):918–926. 10.1001/jama.2017.11470 28898379 PMC5672806

[7] National Comprehensive Cancer Network. NCCN guidelines. 2023. Breast Cancer v 4.2023. https://www.nccn.org/professionals/physician_gls/pdf/breast.pdf

[8] FitzSullivan E , Bassett RL, Kuerer HM, et al. Outcomes of sentinel lymph node-positive breast cancer patients treated with mastectomy without axillary therapy. Ann Surg Oncol. 2017;24(3):652–659. 10.1245/s10434-016-5605-5 PMC5977973 27822630

[9] Mathias BJ , Sun J, Sun W, et al. Surgeon bias in the management of positive sentinel lymph nodes. Clin Breast Cancer. 2021;21(1):74–79. 10.1016/j.clbc.2020.07.010 https://www.sciencedirect.com/science/article/pii/S1526820920301762 32917535

[10] Morigi C , Peradze N, Galimberti V, et al. Feasibility and surgical impact of Z0011 trial criteria in a single-Institution practice. Breast J. 2020;26(7):1330–1336. 10.1111/tbj.13851 32506628

[11] Millen EC , Cavalcante FP, Zerwes F, et al. The Attitudes of Brazilian Breast Surgeons on axillary management in early breast cancer—10 years after the ACOSOG Z0011 trial first publication. Ann Surg Oncol. 2022;29(2):1087–1095. 10.1245/s10434-021-10812-6 34570334

[12] Jain U , Kothari A. Sentinel lymph node biopsy (SLNB) post-NACT and targeted axillary dissection (TAD). In: Chintamani, editor. Sentinel node biopsy in breast cancer. New Delhi: Springer India; 2023:115–124. 10.1007/978-81-322-3994-9_10

[13] Parmar V , Nair NS. Addressing Node-Negative Axilla (N0) in Resource-Constrained Scenarios. In: Chintamani, editor. Sentinel node biopsy in breast cancer. New Delhi: Springer India; 2023:125–131. 10.1007/978-81-322-3994-9_11

[14] Hamdy O , El-Badrawy A, Saleh GA, et al. Preoperative localization of sentinel lymph node in breast cancer patients by silver wire insertion or liquid charcoal injection guided by CT lymphography. Breast J. 2020;26(4):617–624. 10.1111/tbj.13511 31448502

[15] Wong SM , Basik M, Florianova L, et al. Oncologic safety of sentinel lymph node biopsy alone after neoadjuvant chemotherapy for breast cancer. Ann Surg Oncol. 2021;28(5):2621–2629. 10.1245/s10434-020-09211-0. 33095362

[16] Galimberti V , Ribeiro Fontana SK, Vicini E, et al. “This house believes that: Sentinel node biopsy alone is better than TAD after NACT for cN+ patients.”. The Breast. 2023;67:21–25. 10.1016/j.breast.2022.12.010 https://www.sciencedirect.com/science/article/pii/S0960977622002107 36566690 PMC9803818

[17] Barrio AV , Montagna G, Mamtani A, et al. Nodal recurrence in patients with node-positive breast cancer treated with sentinel node biopsy alone after neoadjuvant chemotherapy—a rare event. JAMA Oncol 2021;7(12):1851–1855. 10.1001/jamaoncol.2021.4394 34617979 PMC8498932

[18] Swarnkar PK , Tayeh S, Michell MJ, Mokbel K. The evolving role of marked lymph node biopsy (Mlnb) and targeted axillary dissection (tad) after neoadjuvant chemotherapy (nact) for node‐positive breast cancer: Systematic review and pooled analysis. Cancers. 2021;13(7):1539. 10.3390/cancers13071539 33810544 PMC8037051

[19] Goel N , Yadegarynia S, Rodgers S, et al. Axillary response rates to neoadjuvant chemotherapy in breast cancer patients with advanced nodal disease. J Surg Oncol. 2021;124(1):25–32. 10.1002/jso.26480 33852160

[20] Park TS , Thomas SM, Rosenberger LH, et al. The association of extent of axillary surgery and survival in women with N2-3 invasive breast cancer. Ann Surg Oncol. 2018;25(10):3019–3029. 10.1245/s10434-018-6587-2 29978365 PMC6474911

[21] Sun X , Wang XE, Zhang ZP, et al. Neoadjuvant therapy and sentinel lymph node biopsy in HER2-positive breast cancer patients: results from the PEONY trial. Breast Cancer Res Treat. 2020;180(2):423–428. 10.1007/s10549-020-05559-9 32034581

[22] Leon-Ferre RA , Hieken TJ, Boughey JC. The landmark series: neoadjuvant chemotherapy for triple-negative and HER2-positive breast cancer. Ann Surg Oncol. 2021;28(4):2111–2119. 10.1245/s10434-020-09480-9 33486641 PMC8818316

[23] Polotto S , Rocco N, Catanuto G. Defining an N0 Axilla: pre-SNB assessment of the axilla. In: Chintamani, editor. Sentinel node biopsy in breast cancer. New Delhi: Springer India; 2023:17–38. 10.1007/978-81-322-3994-9_3

